# Analysis of Factors Affecting Hematopoietic Stem Cell Mobilization Efficiency and Early Hematopoietic Reconstruction Indicators during Autologous Peripheral Blood Hematopoietic Stem Cell Transplantation

**DOI:** 10.1055/s-0044-1786006

**Published:** 2024-04-18

**Authors:** Hao Shi, Yaya Duan, Xinting Bu

**Affiliations:** 1 Department of Hematology, Xuzhou Central Hospital, Xuzhou, Jiangsu Province, China

**Keywords:** autologous stem cell transplantation, peripheral blood stem cell, platelet value at the time of collection, megakaryocytic hematopoietic reconstruction

## Abstract

**Purpose** To analyze the factors affecting the mobilization efficiency of hematopoietic stem cells and hematopoietic reconstruction indicators during autologous peripheral hematopoietic stem cell transplantation.

**Methods** The clinical data of 54 patients who underwent autologous peripheral blood hematopoietic stem cell mobilization and transplantation at Xuzhou Central Hospital from May 2016 to April 2023 were retrospectively analyzed. The gender, age, disease type, mobilization regimen, number of chemotherapy sessions, G-CSF (granulocyte colony-stimulating factor) dosage, and platelet number at the time of collection were also collected. Moreover, the relationship between these indicators with mobilization results and hematopoietic reconstruction was analyzed.

**Results** Results showed that age, disease type, and number of collections were significantly related to the mobilization results (number of CD34+ hematopoietic stem cells). Furthermore, multivariate analysis showed that the number of collections was an independent factor affecting mobilization efficiency. Similarly, age, platelet value at the time of collection, CD34+ stem cell value during collection, white blood cell count, and number of chemotherapy times were significantly related to the time of megakaryocytic hematopoietic reconstruction. Multifactor analysis found that age and platelet count were independent factors affecting the reconstruction time of the megakaryocytic system. However, no factor was related to the time of granulocyte hematopoietic reconstruction.

**Conclusion** Platelet count and age when collecting hematopoietic stem cells are closely related to megakaryocytic hematopoietic reconstruction and are key indicators of early hematopoietic reconstruction after autologous hematopoietic stem cell transplantation.

## Introduction

The incidence of malignant hematological diseases, such as lymphoma and multiple myeloma (MM), has been increasing in recent years, thus seriously threatening people's health. Epidemiological statistics have shown that malignant lymphoma ranks ninth among malignant tumors worldwide. MM is common in the blood system and has complex pathogenesis, thus limiting its treatment. Nonetheless, autologous hematopoietic stem cell transplantation (HSCT) is a recommended treatment option for various hematological malignancies, such as malignant lymphoma, MM, and acute leukemia. Autologous HSCT is safe, low cost, and has a short cycle since it does not need a donor, has no strong immunosuppression, and has no graft-versus-host disease. This study aimed to analyze the factors affecting mobilization efficiency and hematopoietic reconstruction of 54 patients who underwent autologous peripheral blood hematopoietic stem cell mobilization and transplantation. Therefore, the study findings may provide a practical basis for clinical treatment of malignant hematological diseases.

## Materials and Methods

### Case Information

A total of 54 patients (26 males and 28 females) who underwent autologous peripheral blood HSCT in our hospital from May 2016 to April 2023 were included in this study. The median age and weight of the patients were 54 years (8–72) and 64 kg (50–70), respectively. A total of 35 patients had lymphoma (5 cases of Hodgkin lymphoma [HL], 30 cases of non-Hodgkin lymphoma [NHL]) and 19 patients had MM ( Table 1 ). Inclusion criteria were lymphoma patients confirmed via pathological results of biopsy specimens; patients with monoclonal plasma tumor cells confirmed via bone marrow cytology, flow cytometry, and bone marrow biopsy, with evidence of bone destruction, indicating MM. Exclusion criteria included patients who did not undergo autologous HSCT or receive CAR-T immunotherapy (new small molecule kinase inhibitors or chimeric antigen receptor T cells).

**Table 1:** General patient information

		Lymphoma			*F* /χ ^2^	*p*
		HL	NHL	MM		
	*n*	5	30	19		
	Age	28.20 ± 9.42	50.23 ± 14.90	55.26 ± 6.37	9.848	<0.001
	Number of chemotherapy sessions	6 (6∼8)	6 (4∼7)	4 (4∼6)	3.178	0.204
	Remission state					
	CR	2	19	14	2.034	0.362
	PR	3	11	5		
Mobilization plan	BEAC	0	6	0	92.25	<0.001
	CTX	0	0	19		
	DHAP	1	1	0		
	ECHOP	3	1	0		
	EPOCH	0	2	0		
	ESHAP	0	3	0		
	G-CSF	1	0	0		
	GDP	0	1	0		
	HyperCVAD	0	6	0		
	R-DA-EPOCH	0	3	0		
	R-ESHAP	0	2	0		
	R-GDP	0	1	0		
	R-ICE	0	1	0		
	R-MTX	0	1	0		
	RECHOP	0	1	0		
	REPOCH	0	1	0		

Abbreviations: G-CSF, granulocyte colony-stimulating factor; HL, Hodgkin lymphoma; NHL, non-Hodgkin lymphoma; MM, multiple myeloma.

### Methods

#### Hematopoietic Stem Cell Mobilization and Collection

Peripheral blood hematopoietic stem cells were mobilized using chemotherapy + G-CSF (granulocyte colony-stimulating factor) regimen or cyclophosphamide + G-CSF or G-CSF alone. Patients in the lymphoma group received CHOPE, (R) ESHAP, EPOCH, DHAP, and HyperCVAD combined with G-CSF as a mobilization program. Patients in the myeloma group received CTX combined with G-CSF, as the mobilization regimen. Furthermore, G-CSF subcutaneous injection (8–10 µg/kg/d) was given every day for more than four consecutive days when the white blood cells (WBCs) dropped to the lowest point. In addition, peripheral blood hematopoietic stem cells were collected (COBE Spectra, U.S. BIS) when the WBC count ≥4 × 10 ^9^ /L, platelet (PLT) count ≥20 × 10 ^9^ /L, the proportion of monocytes increased, and the peripheral blood CD34+ cell content >0.1%. The collection was performed 1 to 2 times based on the CD34+ cell count. Successful mobilization of autologous peripheral blood hematopoietic stem cells was achieved when the number of mononuclear cells in the collected specimen ≥5 × 10 ^8^ /kg and the number of CD34+ cells ≥2 × 10 ^6^ /kg, otherwise mobilization failure occurred.

#### Hematopoietic Stem Cell Freezing and Recovery

The same type of plasma and dimethyl sulfoxide was added to the collected samples (volume fraction; 20% and 10%), then stored in ultralow temperature refrigerator (Sanyo MDF-192N type, Sanyo Co., Ltd., Japan) at −80°C. The patient was given intravenous reinfusion immediately after resuscitation in a 42°C constant-temperature water bath.

#### Pretreatment Plan

Patients in the lymphoma group were pretreated with BuCy and BEAC regimens, whereas those in the myeloma group were pretreated with melphalan (140 mg/m ^2^ for 2 days).

#### Monitoring of Hematopoietic Reconstruction

Routine blood tests every day were conducted after transfusion of stem cells to monitor the neutrophil and PLT number. Successful hematopoietic reconstitution was achieved when neutrophil count was ≥0.5 × 10 ^9^ /L and the PLT count was ≥20 × 10 ^9^ /L.

#### Statistical Analysis

Data analysis was performed using SPSS19.0 software. The measurement data were expressed as mean ± standard deviation, whereas the count data were expressed as numerical values (rates). The measurement data among the three groups were compared using one-way analysis of variance. χ ^2^ test was used to analyze differences in numerical variables among the three groups. Multivariate analysis was performed using linear regression analysis. *p* < 0.05 was considered a statistically significant difference.

## Results

### General Patient Information

A total of 54 patients with hematological malignancies were included in this study, including 5 HL patients, 30 NHL patients, and 19 MM patients ( Table 1 ). The patient ages were significantly different among the three groups. Furthermore, mobilization plans for autologous HSCT were significantly different among the three groups. Notably, HL patients mostly used the ECHOP plan (three cases), followed by the G-CSF plan and the DHAP plan. The MM patients used the CTX mobilization regimen. For NHL patients, six patients used the BEAC regimen, six patients used the HyperCVAD regimen, three patients used the ESHAP regimen, three patients used the R-DA-EPOCH regimen, and two patients used the EPOCH regimen. Furthermore, two NHL patients were treated with the R-ESHAP regimen, whereas the remaining patients were treated with DHAP, ECHOP, GDP, R-GDP, R-ICE, R-MTX, RECHOP, and REPOCH regimens in one case each (R, D, A, G, P, E, C, H, O, I, HA indicate rituximab, dexamethasone, cytarabine, gemcitabine, cisplatin, etoposide, cyclophosphamide, doxorubicin, and vincristine, ifosfamide, and high-dose cytarabine, respectively)

### Characteristics of Patients with Mobilization Failure

Six patients, including three lymphoma and three myeloma patients, failed to mobilize. The lymphoma patients had mantle cell lymphoma (one case) and relapsed diffuse large B-cell lymphoma (one germinal center B cell (GCB) subtype and one non-GCB subtype). The lymphoma cases were in remission. The first myeloma patient had a complex karyotype, IgA-K type, whereas the second myeloma patient had the IgA-L type and had a poor prognosis with 1q21 amplification. The third myeloma patient had L light chain type, complex karyotype, and poor prognosis with 1q21 amplification. The lymphoma types in successfully mobilized patients included HL, follicular lymphoma, diffuse large B-cell lymphoma, mantle cell lymphoma, high-grade lymphoma, anaplastic large cell lymphoma, peripheral T-cell lymphoma, lymphoblastic lymphoma. The myeloma types in successfully mobilized patients included IgG-K, IgG-L, IgA-K, IgA-L, IgD-L, light chain K, and light chain L.

### Stem Cell Mobilization

The mobilization dose of G-CSF, precollection WBC, monocyte proportion, and PLT were not significantly different among the three groups ( Table 2 )

**Table 2:** Mobilization results in each group

			Collection times					
		*n*	1	2	G-CSF dose	WBC	Monocyte proportion	PLT
Lymphoma	HL	5	3	2	2460.0 ± 760.26	42.0 ± 29.66	9.6 ± 7.86	195.2 ± 139.81
	NHL	30	13	17	3346.7 ± 1792.80	28.0 ± 17.44	10.9 ± 8.92	110.3 ± 61.61
	MM	19	1	18	3005.3 ± 1121.74	23.1 ± 13.13	9.7 ± 4.81	128.1 ± 78.22
*F* /χ ^2^			9.894		0.851	2.302	0.174	2.676
*p*			0.007		0.434	0.111	0.841	0.079

Abbreviations: G-CSF, granulocyte colony-stimulating factor; HL, Hodgkin lymphoma; NHL, non-Hodgkin lymphoma; MM, multiple myeloma; PLT, platelet; WBC, white blood cell.

### The Influencing Factors of Mobilization Results (CD34+ Cell Count)

The factors affecting the mobilization results were further analyzed by grouping the influencing factors based on the classification standard or median of the factor. The grouping was performed as follows: G-CSF dose (divided into two groups: >2,800 and ≤2,800 µg), Disease type (divided into three groups: MM, HL, NHL), number of collections (divided into 1 and 2 times), number of chemotherapy before collection (divided into >5 and ≤5 times), PLT count at the time of collection (divided into >100 × 10 ^9^ /L and ≤100 × 10 ^9^ /L), age (divided into >50 and ≤50 years old), remission status at the time of mobilization (divided into complete remission (CR) and partial remission (PR)), WBC count before collection (divided into >22.5 × 10 ^9^ /L and ≤22.5 × 10 ^9^ /L), the proportion of monocytes (divided into >9 and ≤9%). Multifactor grouping was conducted when there was a difference in the number of CD34+ cells in different groups.

**Fig. 1:**
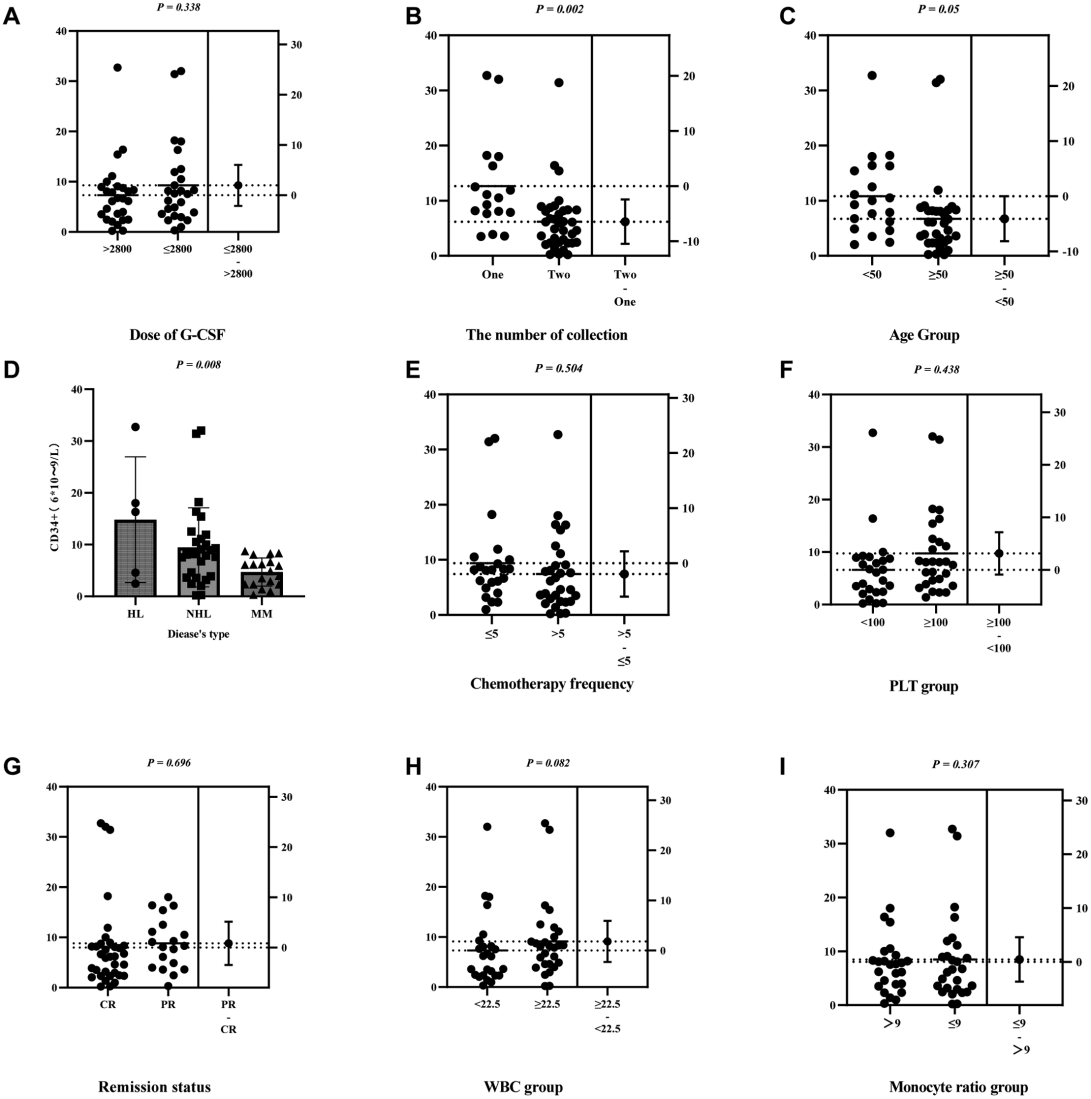
CD34+ cell numbers under different factors. Variable factors which may affect mobilization result were chosen, for example, the abscissa of labels A-I.

The results showed that age, number of collections, and disease type were significantly related to the mobilization results (number of CD34+ hematopoietic stem cells) ( Fig. 1 ). Multifactor linear regression variance results showed that the number of collections was related to the mobilization results at *p* = 0.021 ( Table 3 ).

**Table 3:** Multivariate analysis of factors influencing the number of CD34+ cells after mobilization

Influencing factors	Partial regression coefficient B	Standardized partial regression coefficient β	*T-* value	*p* -Value
Age	−0.513	−0.034	−0.236	0.814
Collection times	−3.365	−0.284	−2.763	0.021
Disease type	−0.368	−0.278	−1.936	0.054

### Hematopoietic Reconstruction Status

A total of 48 patients underwent transplantation. The median time to granulocyte engraftment was 13 days (10–44), whereas the median time to PLT engraftment was 15 days (10–120).

### The Factors Affecting Hematopoietic Reconstruction Indicators

These factors were grouped based on the type or median of the variable. The grouping was performed as follows: total dose of G-CSF (divided into two groups >2,800 and ≤2,800 µg), disease type (divided into three groups: MM, HL, NHL), the number of collections (divided into 1 and 2 times), the number of chemotherapy before collection (divided into >5 and ≤5 times), the PLT count at the time of collection (divided into two groups: >100 × 10 ^9^ /L and ≤100 × 10 ^9^ /L), CD34+ cell count at the time of collection (divided into >8 × 10 ^6^ /kg and ≤8 × 10 ^6^ /kg), age (divided into >50 and ≤50 years old), remission status at the time of mobilization (divided into CR and PR), before collection The number of WBCs (divided into >22.5 × 10 ^9^ /L and ≤22.5 × 10 ^9^ /L), and the proportion of monocytes (divided into >9 and ≤9%).

**Fig. 2:**
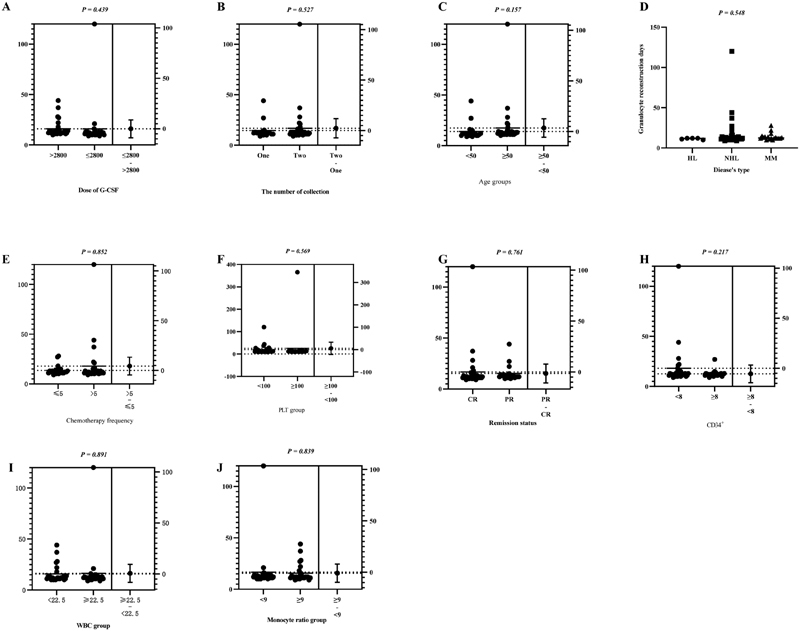
Granulocyte reconstruction time under different groupings. Variable factors which may affect granulocyte reconstitution time were chosen, for example, the abscissa of labels A-J.

Granulocyte reconstitution time was not significantly different among the three groups ( Fig. 2 ), indicating that all patients underwent granulocyte reconstitution. Moreover, disease type, number of chemotherapy, G-CSF dose, and other indicators did not affect the reconstruction time.

### The Effects on Megakaryocytic Cell Reconstruction Time

**Fig. 3:**
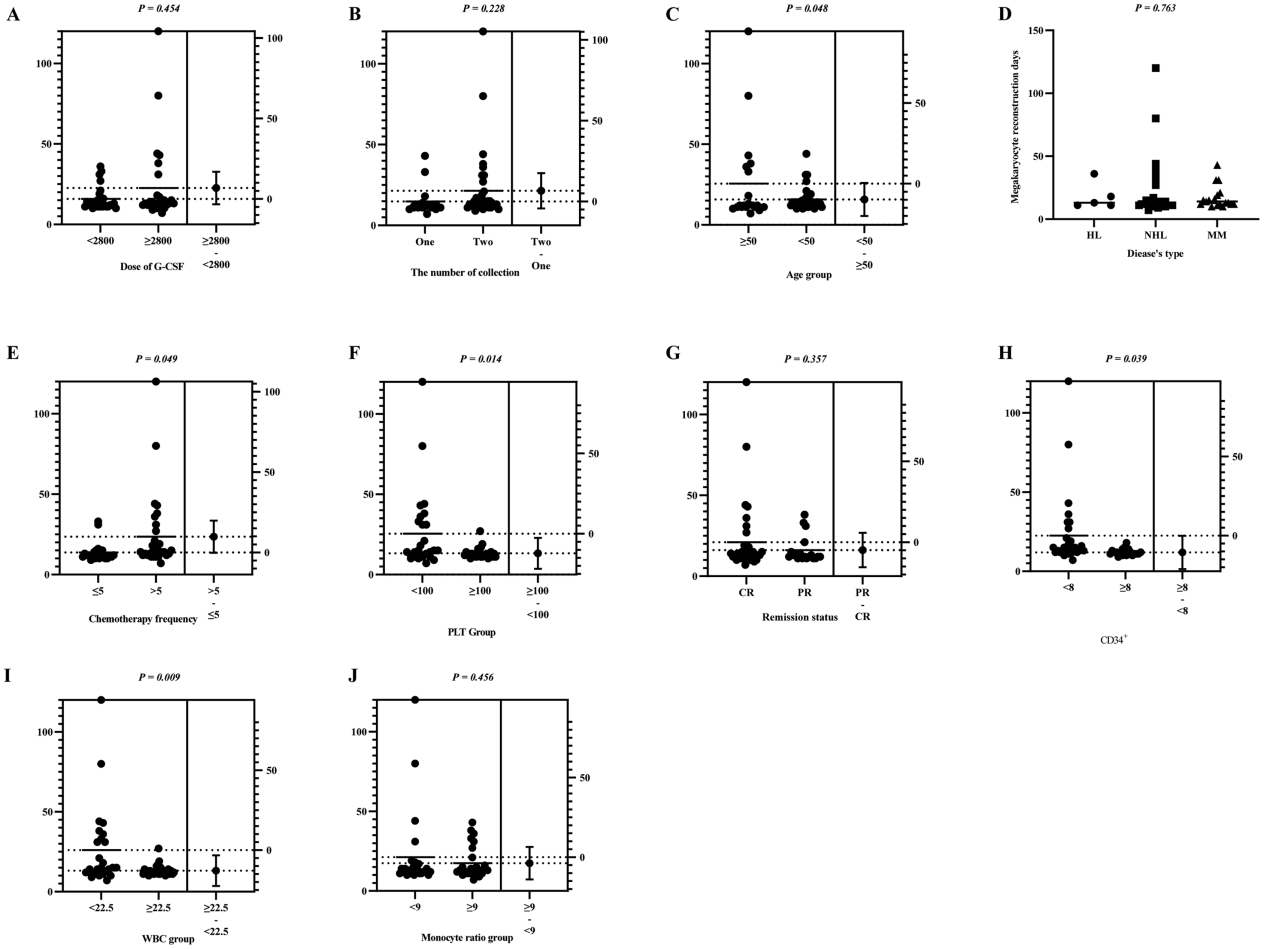
Megakaryocyte reconstruction time under different grouping factors. Variable factors which may affect megakaryocytic reconstruction were chosen, for example, the abscissa of labels A-J.

The grouping was performed as described in the Subsection “The factors affecting hematopoietic reconstruction indicators.” The impact on megakaryocytic cell reconstruction time is shown in Fig. 3 .

The megakaryocyte reconstruction time was significantly different among various age groups, chemotherapy times groups, PLT level groups, CD34+ cell level groups, and WBC level groups, suggesting that these indicators may influence megakaryocyte reconstruction. Multifactor linear regression was further used to analyze the correlation between these indicators and megakaryocyte reconstruction time ( Table 4 ). Results showed that age and PLT level significantly affect the reconstruction time of the megakaryocytic system.

**Table 4:** Multifactor linear regression analysis

Influencing factors	Partial regression coefficient B	Standardized partial regression coefficient β	*T-* value	*p* -Value
Age	0.678	0.510	4.135	<0.001
CD34+ cell count	−0.517	−0.202	−1.662	0.103
Number of chemotherapy sessions	−0.225	0.024	−0.196	0.846
PLT	−0.078	−0.329	−2.617	0.012
WBC	−0.198	−0.189	−1.436	0.158

Abbreviations: PLT, platelet; WBC, white blood cell.

## Discussion

Autologous HSCT is widely used for treating malignant lymphoma and MM. 1 2 Compared with other methods, this transplantation can achieve a higher complete response rate and significantly extend progression-free survival (PFS) and overall survival (OS) time. A prospective (parallel) controlled study of first-generation and second-generation new drugs followed by induction therapy, auto-HSCT, and continuous new drug maintenance therapy showed that the OS of MM patients treated with auto-HSCT was significantly prolonged. Furthermore, auto-HSCT improved PFS, indicating its therapeutic status in newly diagnosed MM patients. 3 Besides, auto-HSCT is crucial for MM patients after transplantation, even if monoclonal antibody-containing regimens are used as induction therapy. 4 Furthermore, auto-HSCT is crucial for relapsed or primary refractory diffuse large B-cell lymphoma or high-grade B-cell lymphoma, refractory mantle cell lymphoma, and peripheral T-cell lymphoma. In summary, auto-HSCT can achieve and maintain remission for most lymphomas and MM.

First, the mobilization and collection of autologous peripheral blood hematopoietic stem cells is a critical step in autologous transplantation. Some case analyzers have identified predictive factors that affect mobilization, such as history of radiotherapy, multiple times, multidrug combination chemotherapy, low PLT count, and low CD34+ cell count in peripheral blood or bone marrow. In addition, chemotherapy combined with G-CSF can be used to predict mobilization failure. 4 Furthermore, anemia and the use of lenalidomide can indicate mobilization failure in patients with MM. Moreover, tissue type, second-line or above treatment regimen, multidrug combination regimen, high-dose methotrexate/alpha glucocytidine regimen, hyper-CVAD-B mobilization regimen, and prolonged application of G-CSF can predict mobilization failure in lymphoma patients. 5 Some studies have also shown that low body weight, poor remission status (stable disease or progressive disease), and low peripheral blood WBC count at the time of collection are significantly related to the CD34+ cell count. 6 Studies have also shown that chemotherapy combined with G-CSF is better than G-CSF alone. 7 Particularly, chemotherapy drugs that damage stem cells, such as nitrogen mustard, methylbenzohydrazide, melphalan, and cytarabine (>7.5 g), are not suitable for mobilization. Besides, the number of chemotherapy treatments cannot exceed 11 times. 8 The Italian Stem Cell Transplantation Working Group also showed that age ≥65 years, bone marrow infiltration, and refractory or progressive disease status can predict mobilization failure in lymphoma patients. 9 In this study, the number of collections was identified as an independent factor related to mobilization. Therefore, most hematopoietic stem cells can be obtained by avoiding multiple chemotherapy treatments, avoiding the application of some drugs, such as lenalidomide, and collecting peripheral hematopoietic stem cells once with an appropriate amount of G-CSF.

Mobilization must follow the principle of individualization, and the timing of stem cell collection should be determined based on the CD34+ cell count ratio in peripheral blood. Previous reports from multiple single centers showed that the mobilization failure rate is about 10.0%–47.5%. 4 5 In this study, the mobilization failure rate was 11.1%. The launch and application of plerixafor in recent years have increased mobilization success rate even in older patients, second-line mobilization, recurrence, or multiple chemotherapy patients. Two multicenter, randomized, double-blinded, placebo-controlled phase III studies recently confirmed that plerixafor combined with G-CSF is superior to G-CSF alone in NHL and myeloma patients. 10 11 Several new hematopoietic stem cell mobilization pathways are currently being explored, including drugs targeting the SDF-1/CXCR4 axis, 12 S1P agonists, 13 and VCAM/VLA-4 inhibitors. 14

Second, hematopoietic reconstruction after transplantation is also the main manifestation of the success of auto-HSCT. Studies have shown that the number of CD34+ cells transfused can predict neutrophil and PLT engraftment. 13 The number of CD34+ cells <1.5∼2.5 × 10 ^6^ /kg may delay the recovery of neutrophils and PLTs, whereas CD34+ cells <1 × 10 ^6^ /kg may cause implantation failure. 14 The minimum CD34+ cell number should be 2 × 10 ^6^ /kg. 7 Some studies have shown that a CD34+ cell number >6 × 10 ^6^ /kg can enable rapid engraftment and long-term maintenance of PLTs. 8 15 Other studies have compared the number of transplanted CD34+ cells (2–4 × 10 ^6^ /kg, 4–6 × 10 ^6^ /kg, and >6 × 10 ^6^ /kg) and showed that the higher the number of CD34+ cells, the higher the number of transplanted CD34+ cells at 100 days, 6 months, and the higher the proportion of patients with PLTs ≥150 × 10 ^9^ /L at 12 months. 15 In this study, the number of CD34+ hematopoietic stem cells were significantly correlated with megakaryocytic hematopoietic reconstruction. This finding also confirms that megakaryocyte production capacity represents the function of hematopoietic stem cells after transplantation;

Age is crucial for the suitability for auto-HSCT. Age is also a predictor of megakaryotic hematopoietic reconstruction. For example, Hasan et al's research showed that the time for megakaryotic hematopoietic reconstruction in the age group <50 years is 1.7 times that of the group ≥50 years. 16 Another study found that clonal hematopoiesis of unknown significance is associated with failure of stem cell mobilization. The genes screened included ASXL, DNMT3A, JAK2, SF3B1, TET2, and TP53 mutations. 17 Clonal hematopoiesis is common among the elderly. However, the age criteria for predicting transplant outcomes are debatable since most patients with MM are old.

Herein, megakaryocyte reconstruction time was different in various leukocyte numbers, but it was not an independent factor influencing megakaryocytic reconstruction. The peripheral leukocyte number is related to the number of CD34+ cells collected, indicating that it can affect megakaryocyte hematopoietic reconstruction. Moreover, monocyte proportions were not significantly different among the groups.

Previous studies have indicated that low PLT count before collection can predict mobilization success. Besides, some studies have suggested that low PLT count before collection are related to megakaryocytic reconstruction. 18 In this study, low PLT count was identified as an independent factor influencing megakaryocytic reconstruction. Low PLT count before collection may indicate decreased differentiation ability of hematopoietic stem cells into megakaryocytic progenitor cells. This could be because multiple, multidrug combination chemotherapy, and certain drugs can damage hematopoietic stem cell function, 8 even after stimulation, such as G-CSF. Herein, although a sufficient number of CD34+ hematopoietic stem cells were collected, the megakaryocytic hematopoietic reconstruction was delayed due to impaired megakaryocytic generation ability. 19

Thrombopoietin (TPO) receptor agonists can increase PLT number in patients with primary immune thrombocytopenia and aplastic anemia by promoting the maturation of the megakaryocytic system. Besides, TPO receptor agonists are widely used for poor PLT engraftment after allogeneic HSCT. 20 The TPO receptor agonist, avatrombopag, combined with mesenchymal stem cells (MSCs). MSCs can safely and effectively treat posttransplant thrombocytopenia 21 for the reconstruction of megakaryocyte hematopoiesis after auto-HSCT. The diagnosis and treatment of malignant hematological diseases are increasingly improving. Therefore, this research may help in the accurate prediction of hematopoietic reconstruction after auto-HSCT, thereby improving the success rate of auto-HSCT.
